# Associação entre fraturas sacrais e lesões de anel pélvico: Estudo correlativo entre as morfologias

**DOI:** 10.1055/s-0046-1820459

**Published:** 2026-06-16

**Authors:** João Victor Jordão Sousa, João Pedro Lemos Soares, Antenor Aguiar Almeida Junior, Tomas Costa Arslanian, Eriko Gonçalves Filgueira

**Affiliations:** 1 Serviço de Ortopedia e Traumatologia, Hospital de Base do Distrito Federal, Brasília, DF, Brasil

**Keywords:** classificação, ossos pélvicos, sacro, classification, pelvic bones, sacrum

## Abstract

**Objetivo:**

Investigar, por meio de coorte histórica, a associação entre fraturas sacrais e lesões do anel pélvico (LAPs), por meio da correlação entre seus subtipos classificatórios.

**Métodos:**

Foram selecionados 45 pacientes internados em um centro traumatológico terciário entre março de 2023 e maio de 2025. Foram avaliados exames de imagem quanto à presença de LAPs e fraturas sacrais. Em seguida, os pacientes foram classificados segundo as classificações de Tile e de Denis. Então, foram submetidos à análise estatística seguindo a metodologia do teste de McNemar–Bowker e para avaliar a força da associação por meio do coeficiente V de Cramér.

**Resultados:**

Na classificação das LAPs por Tile, encontrou-se predominância do tipo B (62,22%), seguido do tipo C (35,56%) e do tipo A (2,22%). Quanto às fraturas sacrais classificadas por Denis, o tipo mais prevalente foi I (50%), seguido do tipo II (28,57%) e do tipo III (21,43%). Ao associar os dados, observou-se que nas LAPs Tile B houve uma incidência de 85,7% de fratura sacral associada, sendo que, destas, 66,6% eram Denis I. Já nas LAPs Tile C, a incidência de fratura sacral associada foi de 100%, sendo 50% descritas como Denis II. Houve associação significativa entre as classificações de Tile e de Denis ( *p* < 0,001), com correlação moderada (V de Cramér: 0,27–0,35).

**Conclusão:**

O estudo sugere correlações entre subtipos de LAP e fraturas sacrais, sendo mais frequentes as associações Tile B-Denis I e Tile C-Denis II. Essa associação pode acelerar a investigação e melhorar os desfechos dos pacientes.

## Abstract (en)

**Objective:**

To investigate, in a retrospective cohort, the association between sacral fractures and pelvic ring injuries (PRIs) by correlating their respective classification subtypes.

**Methods:**

We selected 45 patients admitted to a tertiary trauma center between March 2023 and May 2025. Imaging studies were evaluated for the presence of PRIs and sacral fractures, followed by their classification according to the Tile and Denis systems. Then, a statistical analysis was performed using the McNemar–Bowker test and the Cramér's V coefficient to assess the strength of the association.

**Results:**

Among the PRIs classified according to the Tile system, type B was predominant (62.22%), followed by type C (35.56%), and type A (2.22%). Among the sacral fractures classified according to the Denis system, type I was the most prevalent (50%), followed by type II (28.57%), and type III (21.43%). When the classifications were correlated, Tile type-B PRIs showed an 85.7% incidence of associated sacral fractures, 66.6% of which were Denis type I. For Tile type-C PRIs, the incidence of associated sacral fractures was of 100%, with 50% classified as Denis type II. There was a significant association between the Tile and Denis classifications ( *p* < 0.001), with a moderate correlation (Cramér's V = 0.27–0.35).

**Conclusion:**

The study suggests correlations between PRIs and sacral fracture subtypes, with Tile B-Denis I and Tile C-Denis II representing the most frequent associations. This relationship may facilitate diagnostic investigation and improve patient outcomes.

## Introdução

As lesões do anel pélvico (LAPs) e suas variantes são tema de debates acadêmicos há mais de um século, desde as descrições de Malgaine em 1859, 1 até mais recentemente, com revisões sistemáticas. 2 3

As LAPs abrangem um amplo espectro e apresentam diversas classificações e subtipos. São prevalentes em todas as faixas etárias, sendo responsáveis por aproximadamente 3% de todas as fraturas. Indivíduos na faixa etária de 18 a 44 anos são mais afetados, com predominância do sexo masculino. 4 As rupturas traumáticas do anel pélvico resultam em sua maioria de mecanismos de trauma de alta energia, e representam um importante fator de morbimortalidade. 5 6 7 Na literatura, 2 8 as taxas de mortalidade variam de 10 a 40%, a depender do mecanismo de trauma, da condição clínica prévia do paciente e do manejo terapêutico instituído.

As fraturas sacrais apresentam-se como lesões raras. Contudo, no contexto das LAPs estima-se que 10 a 45% dos casos associam-se a tais fraturas, 9 10 sendo até 30% dos casos não diagnosticados no primeiro atendimento, o que impõe desfechos ruins a esses pacientes. 11 12

Diversas classificações foram criadas a fim de melhor compreender e caracterizar esse grupo de lesões. Entre as classificações, as mais utilizadas dizem respeito à estabilidade do anel pélvico, como a de Tile, 13 e à morfologia da fratura sacral, como a de Denis. 4

Existem estudos que abordam a associação entre fraturas sacrais e lesões de anel pélvico. No entanto, a literatura apresenta-se escassa ao correlacionar os subtipos entre si, na tentativa de evidenciar a incidência dessas lesões sincrônicas. 14 15 16

O objetivo deste estudo é investigar, mediante uma avaliação retrospectiva, a associação entre fraturas sacrais e LAPs, por meio da correlação de seus subtipos.

## Materiais e Métodos

Por meio de uma avaliação retrospectiva, em formato de coorte, realizou-se um amplo levantamento do acervo de um hospital terciário, referência em trauma, em busca de LAPs e fraturas sacrais, de março de 2023 até maio de 2025. Foram identificados 55 casos de LAP e/ou fratura de sacro em pacientes que necessitaram de hospitalização.

Foram aplicados, como critérios de inclusão, a presença de LAPs associadas ou não às fraturas sacrais, de etiologia traumática, bem como a disponibilidade de exames radiográficos (Rx) e tomográficos (tomografia computadorizada, TC) que permitiam a classificação das lesões; como critérios de exclusão, pacientes com estudos complementares incompletos, portadores de etiologia não traumática e com imaturidade esquelética.

Após a triagem, 10 pacientes foram excluídos do estudo, sendo 1 por imaturidade esquelética, 5 por exames complementares incompletos e 4 não localizados no acervo radiológico, restando 45 participantes para análise.

**Fig. 1:**
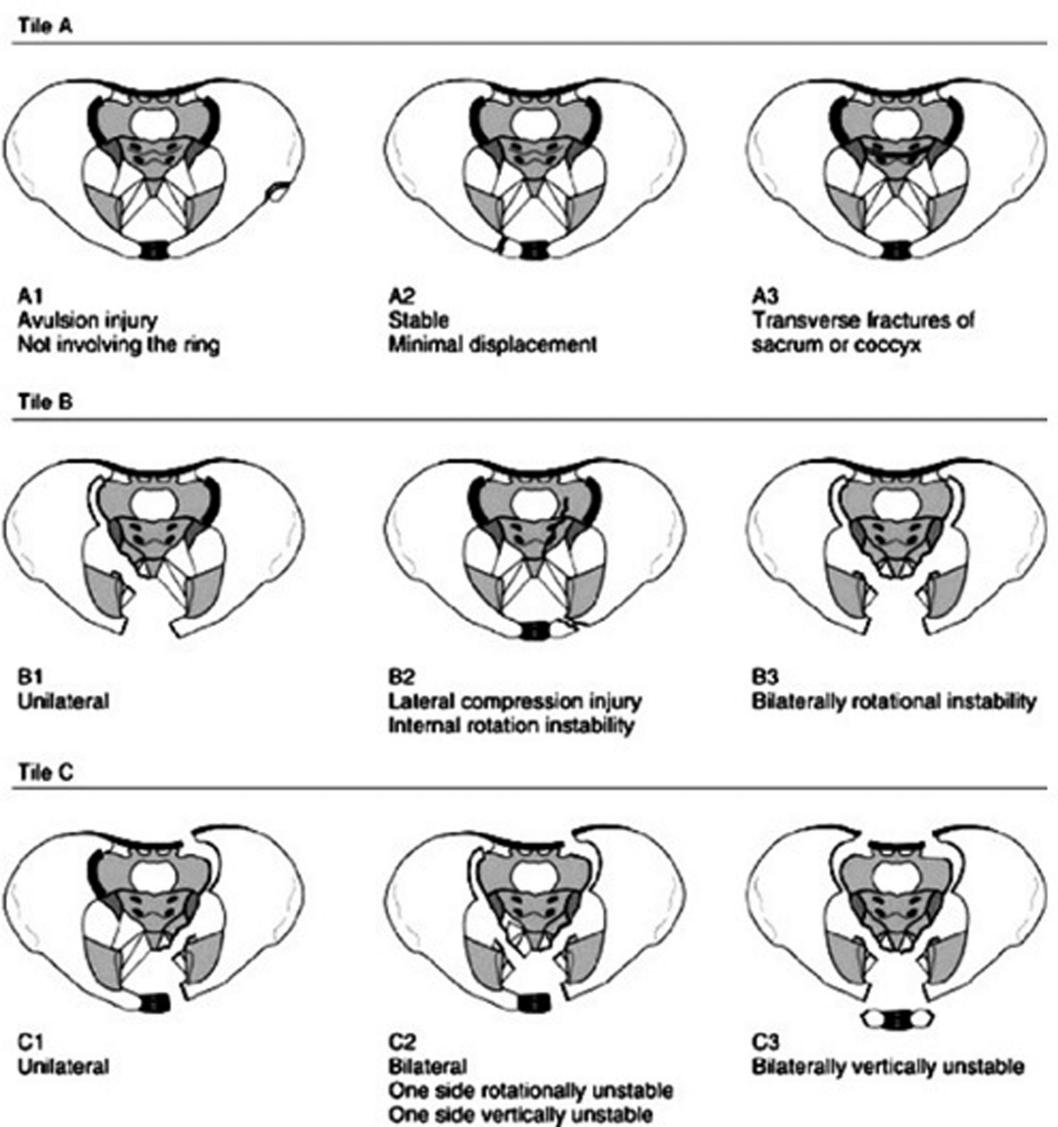
Classificação de Tile para as lesões do anel pélvico.

**Fig. 2:**
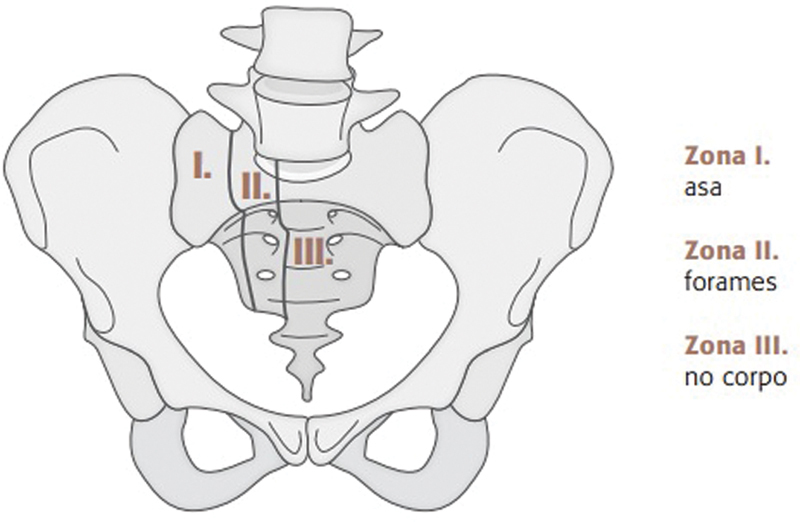
Classificação de Denis para as fraturas sacrais.

Após a seleção dos pacientes, foi realizado o levantamento das imagens de radiografia digital da bacia nas incidências anteroposterior, *inlet* e *outlet* , e tomografias com reconstrução nos planos axial, sagital e coronal, realizadas de forma padronizada no mesmo centro radiológico. Posteriormente, foram avaliadas e classificadas as LAPs seguindo o algoritmo de Tile 13 ( Fig. 1 ), e as fraturas sacrais, seguindo a classificação de Denis ( Fig. 2 ), 11 por quatro examinadores independentes (JPLS, AAAJ, JVJS, TCA, médicos residentes da Unidade de Traumatologia Ortopédica), e casos de foram rediscutidos com um quinto examinador sênior (EGF, cirurgião de trauma ortopédico e coluna vertebral há 25 anos).

Os dados foram armazenados no aplicativo Research Electronic Data Capture (REDcap, Vanderbilt University), para maximizar a segurança e facilitar a análise estatística subsequente.

Realizou-se uma análise descritiva dos dados, com apresentação de medidas de tendência central e de dispersão para as variáveis numéricas, além de frequências absolutas e relativas para as variáveis categóricas (classificações de Tile e Denis). Para avaliar a associação entre essas variáveis, utilizou-se o teste de McNemar–Bowker, com pareamento dos dados em tabela 3 × 3 e posterior submissão à função Bowker do pacote ANSM5 (versão 1.1.1), no programa R (R Foundation for Statistical Computing). A força da associação foi quantificada por meio do coeficiente V de Cramér. O trabalho obteve aprovação do Comitê de Ética institucional, afiliado à Plataforma Brasil, sob o CAAE número 85686924.9.0000.5553.

## Resultados

Foram avaliados 45 pacientes quanto às apresentações radiográfica e tomográfica para as LAPs e fraturas sacrais ( Tabela 1 ).

**Tabela 1:** Casos clínicos

Caso	Idade (anos)	Sexo	Mecanismo de trauma	Local do atendimento	Classificação de Tile (Rx)	Classificação de Tile (TC)	Classificação de Denis (Rx)	Classificação de Denis (TC)
**1**	67	Feminino	Atropelamento	HBDF	C	B	2	2
**2**	74	Feminino	Atropelamento	HBDF	C	C	2	1
**3**	47	Masculino	Atropelamento	HBDF	C	C	2	2
**4**	22	Masculino	Queda de moto	HBDF	B	B	1	1
**5**	23	Feminino	Queda de moto	HBDF	B	B	1	1
**6**	54	Masculino	Atropelamento	HBDF	C	C	1	2
**7**	18	Masculino	Queda de moto	HBDF	C	C	1	2
**8**	56	Feminino	Atropelamento	HBDF	B	B	1	1
**9**	26	Feminino	Queda de plano elevado	HBDF	C	C	2	3
**10**	46	Feminino	Atropelamento	HBDF	A	B	Não identificada fratura	1
**11**	40	Feminino	Atropelamento	HBDF	B	C	2	2
**12**	27	Masculino	Queda de plano elevado	HBDF	A	A	1	1
**13**	56	Masculino	Queda de moto	HBDF	C	C	1	2
**14**	34	Masculino	Queda de plano elevado	HBDF	C	C	2	3
**15**	61	Masculino	Queda de plano elevado	HBDF	C	C	2	3
**16**	NA	Masculino	Acidente automobilístico	HBDF	C	C	2	2
**17**	39	Masculino	Queda de moto	HBDF	B	B	2	2
**18**	56	Masculino	Queda de moto	HBDF	B	B	2	3
**19**	37	Masculino	Atropelamento	HBDF	B	B	1	1
**20**	50	Masculino	Queda de moto	HBDF	C	C	Não identificada fratura	3
**21**	20	Masculino	Atropelamento	HBDF	B	B	1	2
**22**	44	Masculino	Queda de moto	HBDF	B	B	Não identificada fratura	3
**23**	32	Masculino	Atropelamento	HBDF	B	B	2	1
**24**	23	Masculino	Queda de plano elevado	HBDF	B	B	2	1
**25**	84	Masculino	Atropelamento	HBDF	B	B	1	1
**26**	58	Masculino	Queda de moto	HBDF	B	B	1	3
**27**	23	Feminino	Atropelamento	HBDF	C	C	2	1
**28**	23	Masculino	Atropelamento	HBDF	C	C	1	2
**29**	32	Masculino	Acidente automobilístico	HBDF	B	Não identificada fratura	1	1
**30**	61	Feminino	Queda de plano elevado	HBDF	B	B	1	1
**31**	82	Masculino	Atropelamento	HBDF	B	B	Não identificada fratura	Não identificada fratura
**32**	66	Masculino	Queda de plano elevado	HBDF	B	B	1	1
**33**	58	Masculino	Acidente automobilístico	HBDF	B	B	3	3
**34**	63	Masculino	Queda de plano elevado	HBDF	B	B	1	1
**35**	44	Feminino	Acidente automobilístico	HBDF	B	C	1	1
**36**	75	Masculino	Queda de plano elevado	HBDF	C	C	2	2
**37**	56	Masculino	Atropelamento	HBDF	B	B	Não identificada fratura	1
**38**	41	Masculino	Queda de moto	HBDF	B	B	1	1
**39**	35	Masculino	Acidente automobilístico	HBDF	B	B	1	Não identificada fratura
**40**	50	Masculino	Queda de plano elevado	HBDF	B	B	1	1
**41**	19	Feminino	Acidente automobilístico	HBDF	B	B	2	2
**42**	49	Masculino	Atropelamento	HBDF	B	B	1	1
**43**	22	Feminino	Acidente automobilístico	HBDF	B	B	Não identificada fratura	Não identificada fratura
**44**	19	Feminino	Atropelamento	HBDF	B	B	2	1
**45**	26	Masculino	Queda de plano elevado	HBDF	C	C	3	3

Abreviaturas: HBDF, Hospital de Base do Distrito Federal; Rx, radiografia; TC, tomografia computadorizada.

A idade média dos participantes foi de 44 (variação: 18–84) anos. A amostra consistiu em 75,56% (n = 34) de casos do sexo masculino e 24,44% (n = 11) de casos do sexo feminino.

Com relação ao mecanismo de trauma, o atropelamento foi o mais prevalente, com 37,78% (n = 17), seguido da queda de plano elevado, com 24,4% (n = 11), acidente motociclístico, com 22,22% (n = 10), e acidente automobilístico, com 15,56% (n = 7).

**Fig. 3:**
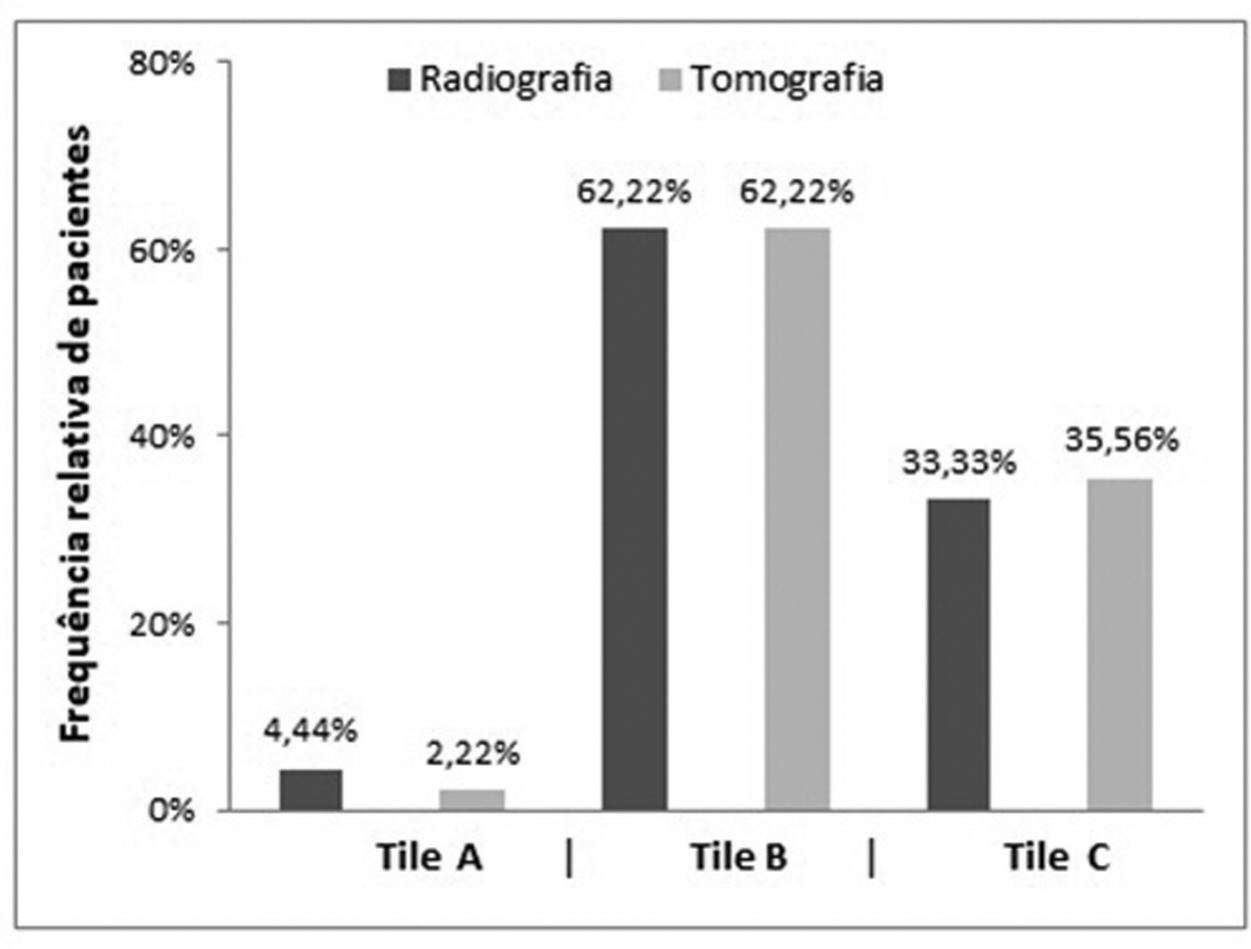
Avaliações radiográfica e tomográfica de lesões do anel pélvico.

Para as LAPs, não se encontrou diferenças com significância estatística entre os resultados para Rx e TC. Observou-se como mais frequente o tipo B de Tile, com62,22% (n = 28), seguido dos tipos C, com 35,56% (n = 16) e A, com 2,22% (n = 1) ( Fig. 3 ).

**Fig. 4:**
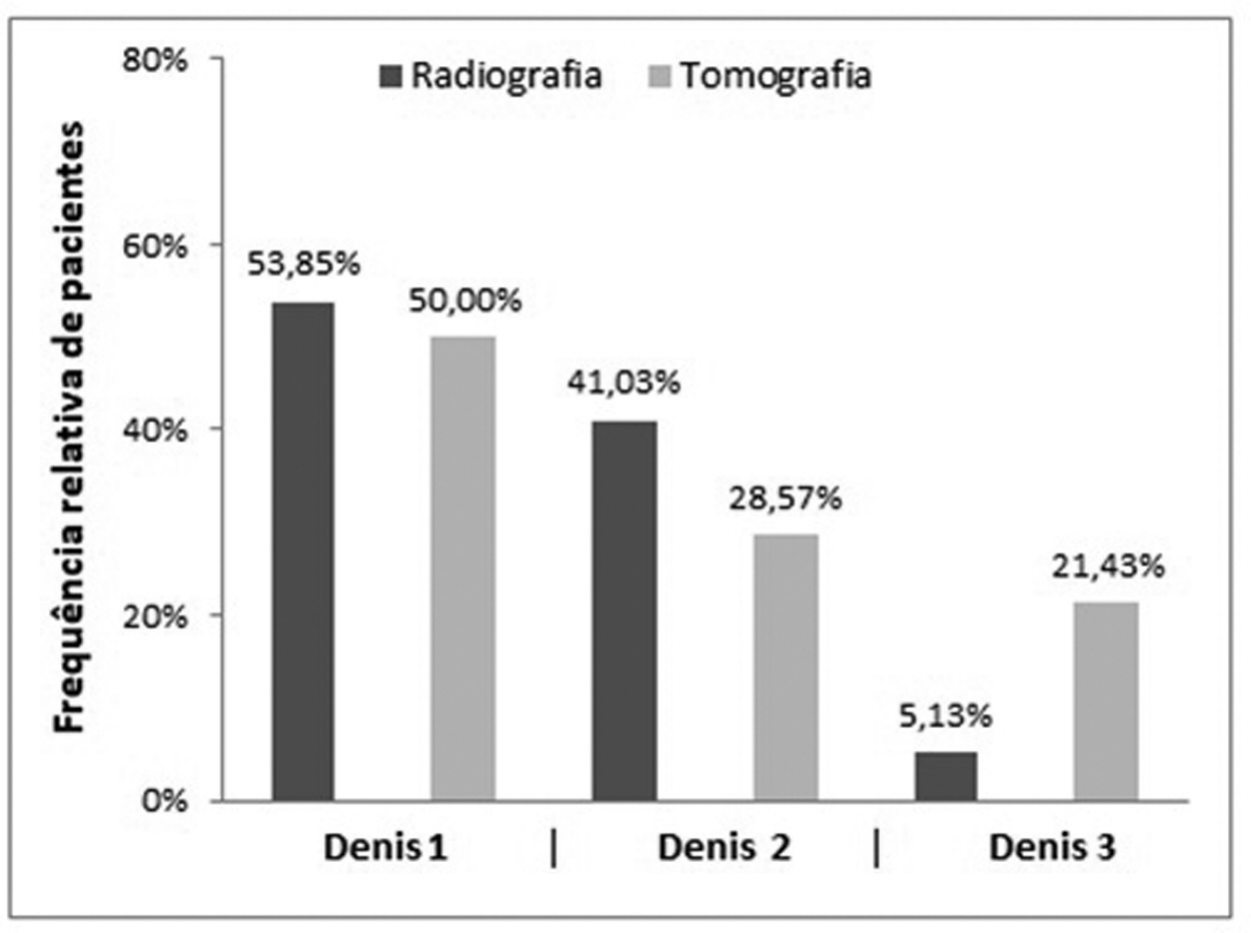
Avaliações radiográfica e tomográfica de fraturas sacrais.

Para as fraturas sacrais, houve divergências relevantes entre Rx e TC. Para a Rx, encontrou-se o tipo I de Denis, com 53,85% (n = 21), seguido dos tipos II, com 41,03%(n = 16) e III, com 5,13% (n = 2), num total de 39 avaliações. Porém, à avaliação tomográfica, observou-se o tipo I, com 50,00% (n = 21), o tipo II, com 28,57% (n = 12), e o tipo III, com 21,43% (n = 9), num total de 42 avaliações ( Fig. 4 ). Em seis avaliações radiográficas, não foram identificadas fraturas sacrais, e somente três desses casos mantiveram-se sem identificação quando realizada a TC. Tais casos foram subtraídos da análise estatística final de suas respectivas metodologias.

Verificou-se uma associação estatisticamente significativa entre as classificações de Tile e Denis, tanto para TC quanto para Rx. Na TC, demonstrou-se McNemar-Bowker χ ^2^_df=3_ = 20.33, *p* < 0.001, com força de associação moderada (V de Cramér = 0.35; tabela 3 × 3). Já na Rx, McNemar-Bowker χ ^2^_df=3_ = 26.4, *p* < 0.001, também com força de associação moderada (V de Cramér = 0.27; tabela 3 × 3).

Nas LAPs Tile B, houve uma incidência de 85,7% de fraturas sacrais associadas, sendo que, destas, 66,6% eram Denis I. Já nas LAPs Tile C, a incidência de fratura sacral associada foi de 100%, sendo 50% descritas como Denis II. Portanto, em ambos os métodos de imagem, observou-se uma concentração relativa de casos de Denis I associados a Tile B, e de Denis II associados a Tile C, conforme indicado na Tabela 2 .

**Tabela 2:** Tabela de contingência

Radiografia				
		***DENIS***		
		*1*	*2*	*3*
***TILE***	*A*	1	0	0
	*B*	16	7	1
	*C*	4	9	1
**Teste de McNemar-Bowker:**	χ ^2^ = 26,4,	*p* < 0,001		
**V de Cramér:**	0,279			
**Tomografia**				
		***DENIS***		
		*1*	*2*	*3*
***TILE***	*A*	1	0	0
	*B*	16	4	4
	*C*	3	8	5
**Teste de McNemar-Bowker:**	χ ^2^ = 20,33,	*p* < 0,001		
**V de Cramér:**	0,352			

## Discussão

As LAPs representam aproximadamente 3% das lesões esqueléticas traumáticas. Até 80% das LAPs ditas instáveis são decorrentes de trauma de alta energia. 3 Estima-se que as fraturas sacrais ocorram em 10 a 45% de todas essas fraturas pélvicas. 9

A primeira classificação reconhecida das LAPs foi descrita por Malgaine em 1859, quando descreveu a *dupla fratura vertical* ; 1 porém, somente um século depois surgiu a primeira classificação abrangente dessas lesões, com a descrição feita por Tile, 13 que classificou as LAPs em estáveis (fraturas do tipo A), rotacionalmente instáveis (tipo B) e verticalmente instáveis (tipo C) 13 ( Fig. 1 ).

As fraturas sacrais são lesões raras que decorrem de traumas de alta energia. A incidência dessa fratura é discutível na literatura 17 devido à dificuldade de identificação na fase aguda. Entre as razões para essas dificuldades encontram-se a limitação na avaliação de imagem pela sobreposição de tecidos moles e gás intestinal na radiografia, e a falha de direcionamento na investigação diagnóstica, haja vista que frequentemente os pacientes não apresentam alterações neurológicas perceptíveis em sua avaliação inicial. 16

Denis et al. 11 classificaram as fraturas sacrais em três tipos, com base em sua localização anatômica ( Fig. 2 ): laterais aos forames (zona I), foraminais (zona II) e mediais aos forames (zona III).

Neste estudo, foi encontrada prevalência de Tile B (62%), Tile C (35%) e Tile A (2,2%), divergentes dos valores encontrados originalmente por Tile. 13 Evidenciou-se também uma prevalência de Denis I (50%), Denis II (28,6%) e Denis III (21,4%). Em um estudo conduzido por Gänsslen et al., 18 identificou-se uma distribuição de 42% (zona 1), 47% (zona 2) e 11% (zona 3), o que diverge dos resultados aqui obtidos. Contudo, estudos 10 que relacionaram o mecanismo de trauma com a fratura sacral encontraram valores distintos para as lesões nas zonas II e III. Esses estudos evidenciaram que, em traumas de compressão anteroposterior, as fraturas sacrais na zona III chegavam a 25% dos casos, o que condiz com os resultados aqui encontrados. Uma associação positiva entre Tile B e Denis I foi caracterizada neste estudo. Levantamentos anteriores corroboraram esta questão, tal qual o de Beckmann e Cai, 19 em que subtipos não instáveis verticalmente apresentaram 48% das lesões na zona I, o que ratifica os dados aqui encontrados.

Evidenciou-se ainda uma associação entre Tile C e Denis II. Estudos prévios 18 19 relataram uma incidência elevada de acometimento das zonas II e III em lesões instáveis verticalmente (Tile C). Tötterman et al. 20 encontraram fraturas na zona II em 72%, e na zona III, em 22% neste perfil de paciente, achados semelhantes aos aqui descritos.

Este estudo tem algumas limitações. Tratando-se de pacientes que necessitaram de hospitalização, em hospital terciário, existe limitação epidemiológica, devido à pequena amostragem de pacientes aptos, apesar de se atingir um valor estatisticamente significativo, bem como uma tendência a pacientes politraumatizados, com casos complexos, de pelves biomecanicamente instáveis.

## Conclusão

As LAPs e as fraturas sacrais apresentam alta morbimortalidade, sendo assim imprescindível o diagnóstico correto e precoce dessas lesões para permitir um manejo assertivo. Conclui-se que existem associações entre subtipos específicos de LAP e morfologias mais prevalentes de fraturas sacrais, tais como Tile B-Denis I e Tile C- Denis II, o que direciona a investigação e auxilia no diagnóstico.
